# Signatures of somatosensory cortical dysfunction in Alzheimer’s disease and HIV-associated neurocognitive disorder

**DOI:** 10.1093/braincomms/fcac169

**Published:** 2022-06-23

**Authors:** Chloe C Casagrande, Alex I Wiesman, Mikki Schantell, Hallie J Johnson, Sara L Wolfson, Jennifer O’Neill, Craig M Johnson, Pamela E May, Susan Swindells, Daniel L Murman, Tony W Wilson

**Affiliations:** Institute for Human Neuroscience, Boys Town National Research Hospital, Boys Town, NE, USA; McConnell Brain Imaging Centre, Montreal Neurological Institute, McGill University, Montreal, QC, Canada; Institute for Human Neuroscience, Boys Town National Research Hospital, Boys Town, NE, USA; College of Medicine, University of Nebraska Medical Center, Omaha, NE, USA; Institute for Human Neuroscience, Boys Town National Research Hospital, Boys Town, NE, USA; Geriatrics Medicine Clinic, University of Nebraska Medical Center, Omaha, NE, USA; Department of Internal Medicine, Division of Infectious Diseases, University of Nebraska Medical Center, Omaha, NE, USA; Department of Radiology, University of Nebraska Medical Center, Omaha, NE, USA; Department of Neurological Sciences, University of Nebraska Medical Center, Omaha, NE, USA; Department of Internal Medicine, Division of Infectious Diseases, University of Nebraska Medical Center, Omaha, NE, USA; Department of Neurological Sciences, University of Nebraska Medical Center, Omaha, NE, USA; Memory Disorders and Behavioral Neurology Program, University of Nebraska Medical Center, Omaha, NE, USA; Institute for Human Neuroscience, Boys Town National Research Hospital, Boys Town, NE, USA; College of Medicine, University of Nebraska Medical Center, Omaha, NE, USA; Department of Pharmacology and Neuroscience, Creighton University, Omaha, NE, USA

**Keywords:** oscillations, gamma, magnetoencephalography, spontaneous activity, dementia

## Abstract

Alzheimer’s disease is the most common type of dementia in the general population, while HIV-associated neurocognitive disorder is the most common neurological comorbidity in those infected with HIV and affects between 40 and 70% of this population. Both conditions are associated with cognitive impairment and have been associated with aberrant functioning in sensory cortices, but far less is known about their disparate effects on neural activity. Identifying such disparate effects is important because it may provide critical data on the similarities and differences in the neuropathology underlying cognitive decline in each condition. In the current study, we utilized magnetoencephalography, extensive neuropsychological testing and a paired-pulse somatosensory gating paradigm to probe differences in somatosensory processing in participants from two ongoing magnetoencephalography studies. The resulting participant groups included 27 cognitively normal controls, 26 participants with HIV-associated neurocognitive disorder and 21 amyloid biomarker-confirmed patients with Alzheimer’s disease. The data were imaged using a beamformer and voxel time series were extracted to identify the oscillatory dynamics serving somatosensory processing, as well as the amplitude of spontaneous cortical activity preceding stimulation onset. Our findings indicated that people with Alzheimer’s disease and HIV-associated neurocognitive disorder exhibit normal somatosensory gating but have distinct aberrations in other elements of somatosensory cortical function. Essentially, those with Alzheimer’s disease exhibited accentuated neural responses to somatosensory stimulation, along with spontaneous gamma activity preceding stimulus onset. In contrast, those with HIV-associated neurocognitive disorder exhibited normal responses to somatosensory stimulation but had sharply elevated spontaneous gamma activity prior to stimulus onset. These distinct aberrations may reflect the impact of different neuropathological mechanisms underlying each condition. Further, given the differential pattern of deficits in somatosensory cortical function, these measures may function as unique biomarkers in each condition and be useful in identifying persons with HIV who may go on to develop Alzheimer’s disease.

## Teaser

Casagrande *et al*. found distinct signatures of aberrant somatosensory processing in participants with Alzheimer’s disease and HIV-related cognitive impairment relative to controls using a somatosensory stimulation paradigm and magnetoencephalographic imaging. Those with Alzheimer’s disease showed stronger oscillatory gamma responses, while those with HIV exhibited elevated spontaneous activity during the baseline.

## Graphical Abstract

**Graphical abstract:**
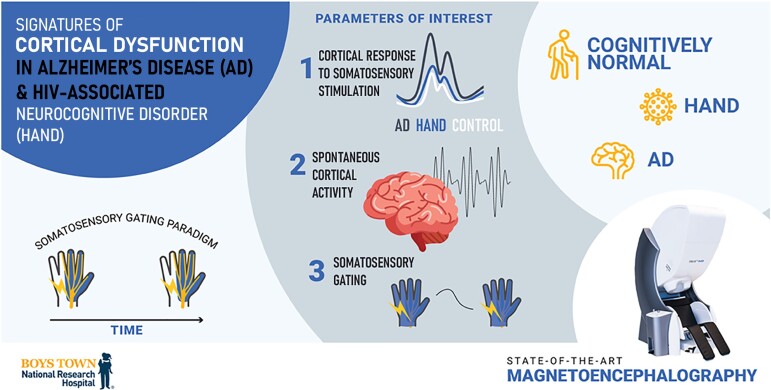

## Introduction

An estimated 50 million people are diagnosed with dementia worldwide.^1^ Alzheimer’s disease accounts for 60-70% of all dementia cases,^1^ with as many as 10% of Americans 65 and older on the Alzheimer’s disease spectrum (i.e. mild cognitive impairment or Alzheimer’s disease).^2^ The impact of Alzheimer’s disease on cognition and functional independence is profound, with most patients progressing to the point of full functional dependence within a decade of diagnosis. While not as common as Alzheimer’s disease, HIV-associated neurocognitive disorder (HAND) can range from mild cognitive decline with limited impact on daily life to HIV-associated dementia (HAD), where individuals are unable to complete daily tasks independently. Despite the advent of combination antiretroviral therapy (cART), HAND still affects 40–70% of persons with HIV (PWH), although generally these deficits are on the milder side and cases of HAD are relatively rare.^3–8^

While HAND has been widely associated with somatomotor dysfunction,^9–17^ the impact of Alzheimer’s disease on the primary motor and somatosensory cortices is less understood, with some studies suggesting these cortices are spared until late in the disease process.^18–21^ This is surprising, as there is significant acetylcholinergic disruption in Alzheimer’s disease,^22–24^ which could particularly affect long-range axonal projections to pyramidal neurons in Layers 2/3 and 5 and/or inhibit activity in Layer 4 spiny neurons in the somatosensory cortices, thereby disrupting excitatory/inhibitory balance and feedback regulation.^25,26^ Amyloid-beta plaques and hyperphosphorylation have also been linked to abnormal gamma oscillatory activity, which is thought to be largely mediated by GABAergic circuits.^27–30^ Most of the somatomotor studies focusing on HAND have used functional imaging measures whereas those in Alzheimer’s disease have been based on structural imaging methods (i.e. have examined atrophy in these cortical regions). Further, to date, many of the somatomotor studies of patients with Alzheimer’s disease have not focused on biomarker-confirmed patients (e.g. amyloid-positive patients). Although research examining the potential commonalities between HAND and Alzheimer’s disease is in its infancy, it is speculated that neurodegeneration in both conditions may arise from persistent neuroinflammation.^31–34^ Thus, research examining the differences and commonalities could inform the neuropathological mechanisms underlying both conditions and help derive markers to distinguish Alzheimer’s disease from other forms of dementia (e.g. frontotemporal, HAD, etc.), as well as HAND from early Alzheimer’s disease in older PWH.

Sensory gating is a neurophysiological phenomenon whereby a diminished neural response to the second stimulus in an identical pair of stimuli is observed. It is thought to reflect the ability of the CNS to inhibit irrelevant and redundant information thereby reserving cognitive resources for the processing of more behaviourally relevant stimuli.^35^ Sensory gating is generally understood as a bottom-up inhibitory process, and an extensive literature has shown robust gating of gamma-frequency oscillations.^13,15,36–39^ While numerous studies have found aberrant auditory gating in Alzheimer’s disease, somatosensory gating has been examined only once in Alzheimer’s disease^40^ and only a handful of times in HAND.^13–15^ To the best of our knowledge, no study to date has compared somatosensory gating deficits in participants with HAND versus those with Alzheimer’s disease. Thus, whether differences in gating and somatosensory processing more broadly can distinguish those with Alzheimer’s disease and HAND remains unknown.

In the current study, we utilized the high spatiotemporal resolution of magnetoencephalography (MEG) and a paired-pulse electrical stimulation paradigm that is known to elicit strong somatosensory neural responses in the postcentral gyrus. Thus, we used MEG imaging to derive functional measures of somatosensory processing to identify disease-specific aberrations in people with Alzheimer’s disease and those with HAND relative to their healthy aging peers. Based on the literature examining these groups in isolation, we hypothesized that the HAND group would show deviations in spontaneous power such that people with HAND would exhibit increased spontaneous power relative to the other two groups. Likewise, we hypothesized that both patient groups would exhibit significantly decreased gamma oscillations relative to controls in response to the two stimulations.

## Materials and methods

### Participants

Adult participants (age range: 51–73 years) were drawn from two ongoing MEG studies; one examining healthy and pathological aging in the context of HIV infection (R01-MH103220) and another examining structural and functional aberrations in Alzheimer’s disease (R01-MH116782-S1). Participants were selected for possible inclusion for analysis based upon their completion of a paired-pulse MEG paradigm, 3T structural MRI, demographics and cognitive testing profile. The exclusionary criteria for the two larger studies included any medical illness that affected CNS function (other than HIV or Alzheimer’s disease), neurological disorders (other than HAND or Alzheimer’s disease), psychiatric disease, a history of head trauma and current substance abuse. All persons with HAND were currently receiving effective cART and had undetectable viraemia at the time of enrolment. Viral suppression was determined as <50 copies/mL. Following a full description of the study, written informed consent was obtained according to the guidelines of the University of Nebraska Medical Center’s Institutional Review Board, which approved the study protocols, and all protocols were in accordance with the Declaration of Helsinki.

### Group characterization

Prior to study screening, all participants in the Alzheimer’s disease group were determined to have mild probable Alzheimer’s disease by a fellowship-trained neurologist using standard clinical criteria.^41^ To be included in the final AD group, participants were required to have a positive whole-brain quantitative amyloid-beta [Aβ] PET scan. To be included in the final HAND group, participants with HIV were required to meet the Frascati criteria for HAND as determined by a board-certified clinical neuropsychologist with HIV experience.^42^ For controls, a demographically matched group of cognitively normal healthy older adults, as assessed by a neuropsychological battery, who reported no subjective cognitive concerns nor HIV infection was enrolled. The control group was demographically matched to the patient groups based upon ethnicity, sex, handedness and weight. The final group consisted of 74 participants, including 27 controls, 21 amyloid-PET confirmed patients with Alzheimer’s disease and 26 patients with HAND. With the exceptions of age and education, which were included as covariates of no interest in all analyses, the demographic profile of the three groups were very similar and are described in Table 1.

**Table 1:** Demographics table

	Controls	Alzheimer’s disease	HAND	Significance
Age (years)	64.5 (6.99)	67.1 (6.51)	58.1 (5.99)	*P* = 0.001
Education (years)	16.7 (2.60)	15.3 (2.73)	12.9 (2.15)	*P* = 0.001
Sex (M/F)	15 M, 12 F	7 M, 14 F	14 M, 12 F	N/S
Handedness (R/L)	24 R, 3 L	18 R, 3 L	25 R, 1 L	N/S
Ethnicity (H/NH)	27 NH	21 NH	26 NH	N/S
Weight (kg)	89.1 (23.6)	80.5 (15.0)	81.6 (20.6)	N/S
CD4 Nadir (cells/μL)	—	—	203 (150)	—
Current CD4 (cells/μL)	—	—	755 (385)	—
Time on ART (years)	—	—	11.1 (6.43)	—

Values are displayed as mean (standard deviation) unless otherwise noted.

N/S, not significant at *P* = 0.05; HAND, HIV-associated neurocognitive disorder; M, male; F, female; R, right; L, left; H, Hispanic; NH, non-Hispanic; ART, combination antiretroviral therapy.

### Neuropsychological testing

The cohorts underwent robust neuropsychological assessment using partially overlapping neuropsychological test batteries (see Table 2). Per the Frascati criteria,^42^ or guidelines for identifying HAND, at least five cognitive domains were assessed, including tests of *learning*, *memory*, *attention, executive function*, *motor* and *processing speed*. The Alzheimer’s disease cohort completed a similar comprehensive neuropsychological test battery including tests of *learning, memory*, *attention and executive function*, *language* and *processing speed*. Mean [standard deviation (SD)] data for all neuropsychological tests are provided in Supplementary Tables 1 and 2. In addition, we measured *premorbid function* and *functional impairment* in all participants, along with *general cognitive status* in the Alzheimer’s disease group. Raw scores for tests comprising the batteries in each cohort were demographically corrected using published normative data,^43–48^ and the resulting *z*-scores were averaged among tests within in each respective domain for participant group classification.

**Table 2:** Neuropsychological tests

	Controls	Alzheimer’s disease	HAND	Significance
HVLT learning	0.307 (0.786)	−2.70 (0.534)	−1.68 (0.875)	*P* < 0.0001
HVLT delayed recall	0.167 (0.817)	−2.97 (0.421)	−1.62 (0.906)	*P* < 0.0001
Trail making test, Part A	0.567 (0.832)	−1.93 (1.49)	−0.350 (0.936)	*P* < 0.0001
Trail making test, Part B	0.807 (0.807)	−1.74 (1.23)	−0.165 (0.789)	*P* < 0.0001
FAS test	−0.0741 (0.962)	−1.17 (1.09)	−0.612 (1.06)	*P* = 0.003
Animal naming	0.111 (1.04)	−2.59 (1.35)	−0.562 (1.07)	*P* < 0.0001
WRAT-4 word reading	0.804 (0.891)	−0.170 (0.927)	−0.743 (0.982)	*P* < 0.0001

Values are displayed as mean (standard deviation) unless otherwise noted. Values for neuropsychological assessments are *z*-scores.

HAND, HIV-associated neurocognitive disorder; HVLT, Hopkins verbal learning test; WRAT, wide range achievement test.

### Experimental paradigm

Participants were seated in a nonmagnetic chair with their head positioned within the MEG helmet’s sensor array. Electrical stimulation was delivered to the right median nerve through external cutaneous stimulators connected to a Digitimer DS7A constant-current stimulator system (Digitimer Limited, Letchworth Garden City, UK). At least 80 paired-pulse trials were administered to each participant with an interstimulus interval (ISI) of 500 ms. This ISI between the pulses was chosen based upon data from previous studies.^37,38,49,50^ The interpair interval varied randomly between 4500 and 4800 ms. Each pulse consisted of a 0.2 ms constant-current square wave that was delivered at 10% above the motor threshold required to elicit a slight twitch of the thumb of the right hand.

### MEG data acquisition and structural MRI coregistration

All MEG recordings were performed in a one-layer magnetically shielded room with active shielding engaged for environmental noise compensation. Neuromagnetic responses were sampled continuously at 1 kHz using an acquisition bandwidth of 0.1–330 Hz and an Elekta/MEGIN system (Helsinki, Finland) equipped with 306 magnetic sensors, including 204 planar gradiometers and 102 magnetometers. Participants were monitored throughout data acquisition with a real-time audio–video feed from inside of the magnetically shielded room. MEG data were corrected for head motion, and noise reduction was applied using the signal-space separation method with a temporal extension.^51^ All structural MRI (sMRI) data were aligned parallel to the anterior and posterior commissures and transformed into standardized space. Participants’ MEG data were coregistered with their individual T1-weighted sMRI data prior to source space analyses using Brain Electrical Source Analysis (BESA) MRI (Version 2.0). Following beamformer analysis, each participant’s functional images were also transformed into standardized space using the transform previously applied to the sMRI volume and spatially resampled.

### MEG preprocessing, time-frequency transformation and sensor-level statistics

Cardiac and blink artefacts were removed from the data using signal-space projection. The projection operator was accounted for during the source reconstruction.^52^ Epochs were of 3700 ms duration, including a −700 to −300 ms baseline window (onset of stimulation one occurred at 0 ms). Of note, the baseline was shifted away from the period directly preceding stimulus onset to avoid possible contamination by any anticipatory responses, although there was no evidence of such anticipatory responses in our final analyses. Artefact rejection was based upon a fixed threshold method supplemented with visual inspection. On average, 76.6 (SD 3.47) trials per participant were retained for the final analysis. The average number of trials did not differ by group.

Artefact-free epochs were transformed into the time-frequency domain using complex demodulation.^53–55^ The derived spectral power estimations per sensor were then averaged over trials to generate time-frequency plots of mean spectral density and normalized using the respective bin’s baseline power calculated as the mean power during the −700 to −300 ms period. The specific time-frequency windows used for subsequent source imaging were determined by statistical analysis of the sensor-level spectrograms across all participants and the entire array of gradiometers.

To reduce the risk of false-positive results while maintaining reasonable sensitivity, a two-stage procedure was followed to control for Type 1 error. In the first stage, paired-sample *t*-tests against baseline were conducted on each data point and the output spectrogram of *t*-values was thresholded at *P* < 0.05 to define time-frequency bins containing potentially significant oscillatory deviations across all participants. In stage two, the time-frequency bins that survived the threshold were clustered with temporally and/or spectrally neighbouring bins that were also above the threshold (*P* < 0.05), and a cluster value was derived by summing the *t*-values of all data points in the cluster. Nonparametric permutation testing was then used to derive a distribution of cluster values and the significance level of the observed clusters (from Stage 1) were tested directly using this distribution.^56,57^ For each comparison, 10 000 permutations were computed to build a distribution of cluster values. The time-frequency bins containing significant responses following permutation testing were selected for beamformer analysis (see below). Additional details of the methodology and processing pipeline can be found in recent papers.^14,58^

### MEG beamformer imaging and voxel-based time series

Cortical regions were imaged through the dynamic imaging of coherent sources beamformer,^59,60^ which employs spatial filters in the time-frequency domain to calculate source power for the entire brain volume. The resulting functional images reflect noise-normalized power differences (i.e. active versus passive) per voxel. MEG preprocessing and imaging were conducted in the BESA (Version 7.0) software. Normalized source power was computed for the selected time-frequency periods (see Results) over the entire brain volume per participant at 4.0 × 4.0 × 4.0 mm resolution. The resulting beamformer images were then averaged across all participants to assess the neuroanatomical basis of the significant oscillatory responses identified through the sensor-level analysis.

Voxel time series were then extracted from each participant’s data using the peak voxel coordinates derived from the grand-averaged functional image. To compute virtual sensors, we applied the sensor weighting matrix derived through the forward computation to the preprocessed signal vector, which yielded a time series for the specific coordinate in source space. Note that virtual sensor extraction was completed per participant once the coordinates of interest were known. Using these virtual sensor time series, we computed the spectral power envelope of the frequency bin used in the beamformer analysis, resulting in relative (baseline-corrected) and absolute time series for each participant.

### Statistics

General linear models were first computed on demographic variables to identify those that would need to be controlled for potential group differences in further analyses. Once we identified these variables (i.e. age and education), we then computed general linear models for evaluation of group differences in somatosensory processing, including response amplitude and prestimulation spontaneous activity, and inhibitory function (i.e. sensory gating). To identify group-specific differences, we computed ANOVA models with age and education as covariates of no interest and followed up significant findings with *post hoc* tests to identify the source of any ANOVA effects. Statistical models excluded participants listwise with responses above or below 2.5 SDs of the mean. All statistical tests were two-tailed with an alpha level of 0.05 and were performed in SPSS (Version 25, Armonk, NY, USA).

### Data availability statement

The data that support the findings of this study are available from the corresponding author upon reasonable request.

## Results

All 74 participants successfully completed the MEG and MRI portions of the study. These participants differed in age (*P* = 0.001) and education (*P* = 0.001) but were matched on sex (*P* = 0.258), ethnicity (*P* = 0.999) and weight (*P* = 0.274). Demographic means and SD per group are provided in Table 1. As stated above, age and education were used as covariates in all statistical models. For the persons with HAND, current CD4 count, CD4 nadir and duration of antiretroviral therapy were also collected at the time of the neuropsychological testing session and are reported in Table 1.

### Sensor- and voxel-level analyses

**Figure 1:**
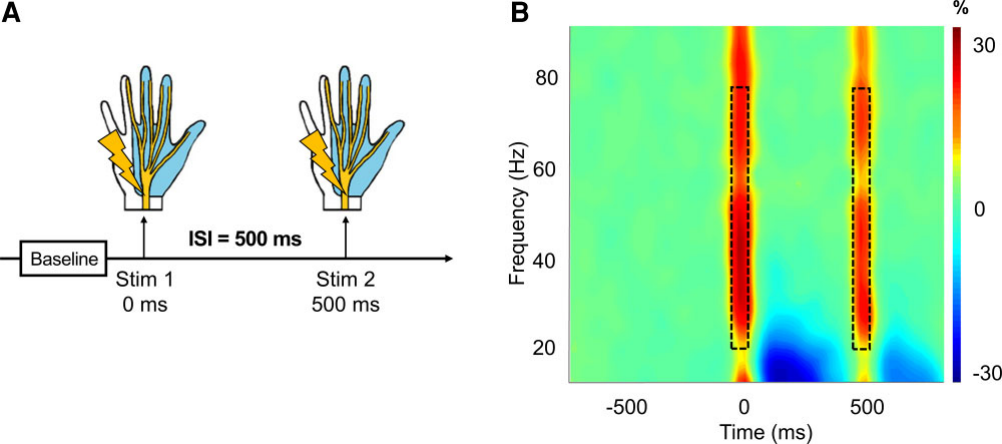
**Task design and time-frequency spectrogram.** (**A**) Overall task design; each epoch was comprised of a baseline (–700 to –300 ms) relative to the onset of the first stimulation at time = 0 ms. The paired-pulse stimulation occurred with an ISI of 500 ms, such that the second stimulus occurred at time = 500 ms. Electrical cutaneous stimulation was applied to the right median nerve which elicits a slight thumb twitch. The light blue region of the hand shows the area served by the right median nerve. (**B**) Spectrogram displaying time-frequency information from a representative gradiometer found over the left sensorimotor strip. The *x*-axis represents time (in milliseconds), and the *y*-axis represents frequency (in Hz). The paired-pulse stimulations occurred at 0 ms (Stimulation 1) and 500 ms (Stimulation 2). The colour bar represents the amplitude threshold (percent change relative to the prestimulus baseline period). Warmer colours indicate an increase in amplitude relative to baseline.

**Figure 2:**
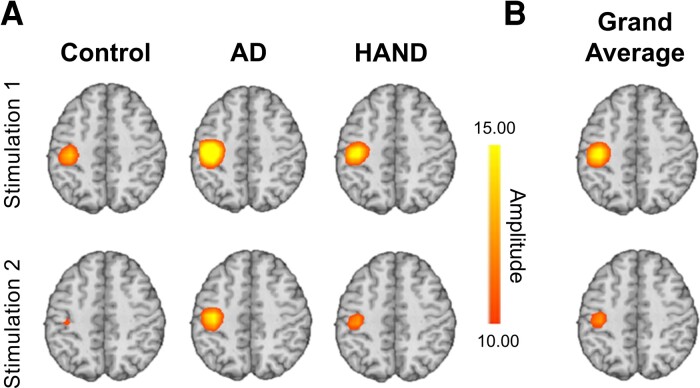
**Beamformer images showing peak somatosensory activity in the left postcentral gyrus.** (**A**) Group averaged images per stimulation. All groups exhibited peak responses in spatially consistent regions of the somatosensory cortex and clear somatosensory gating, although there were group differences observed in the amplitude of the responses. The colour bar embedded shows amplitude thresholds in pseudo *t*-values as applicable to all images in **A** and **B**. The warm colours indicate a neural synchronization event. (**B**) The peak somatosensory responses averaged across all participants. Of note, the response to Stimulation 1 is far more robust than that of Stimulation 2, indicating a gating effect, with the peak in the left postcentral gyrus.

We observed robust broadband synchronizations traversing 20–75 Hz in multiple sensors near the hand region of the left postcentral gyrus that extended temporally to approximately 50 ms after the onset of each stimulus (Fig. 1; cluster-based permutation test: 10 000 permutations, *P* < 0.001, corrected). The resulting images were grand averaged within-group for visualization purposes, and similar peaks were observed across all three groups centred in the left postcentral gyrus directly posterior to the motor hand knob feature of this region (Fig. 2A). The individual beamformer images were grand averaged across all participants and stimulations to derive the peak voxel for virtual sensor analyses (Fig. 2B).

### Virtual sensor analyses reveal Alzheimer’s disease-specific aberrations in somatosensory processing

**Figure 3:**
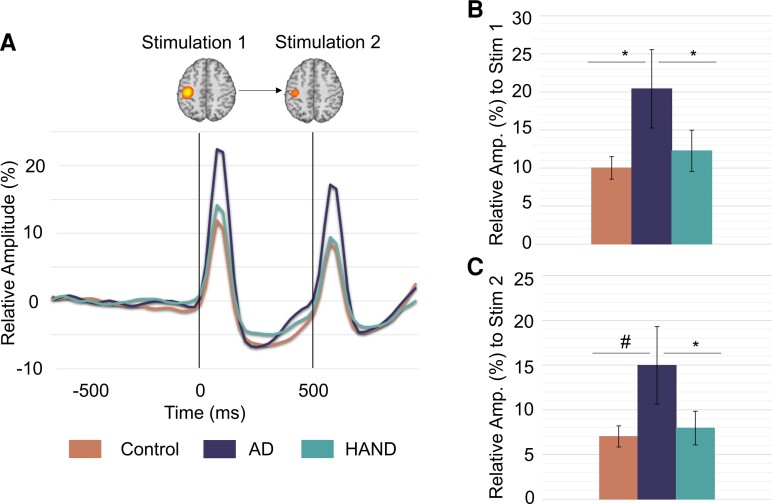
**Virtual sensor time series indicate group differences in oscillatory responses.** (**A**) Relative amplitude time series from the peak voxel across all participants showing neural responses to the stimulation in each group. The colour legend appears on the bottom of the figure. As shown, persons with Alzheimer’s disease exhibited much stronger oscillatory responses to each stimulation compared with both controls and those with HAND. Grand average beamformer images shown above time series reflect location of peak voxel. (**B**) Bar graph of the mean relative amplitude of neural responses to Stimulation 1 at the peak voxel. Data have been averaged over the time window used for beamformer image computation (i.e. 0–50 ms) per participant and then group. Responses were much stronger in those with Alzheimer’s disease. (**C**) Same as **B** except that the data reflect responses to Stimulation 2 (i.e. 500–550 ms). ANOVA models (group × stimulation) were computed, and these indicated significant group differences. Follow-up *post hoc* analyses were conducted. *P < .05; ^#^*P* = 0.054.

We next extracted virtual sensor data from this peak and computed relative power over the 20–75 Hz frequency range. To identify the impact of Alzheimer’s disease and HAND pathologies on somatosensory response amplitude to each stimulation, ANOVA models (group *×* stimulation) were computed, and these indicated significant group and stimulation main effects (*P* < 0.05). To determine where the group differences were arising, we performed *post hoc* testing which indicated that people with probable Alzheimer’s disease differed from controls (*F*(1,45) = 4.295, *P* = 0.044) and people with HAND (*F*(3,44) = 3.310, *P* = 0.036) in the relative power of their responses to Stimulation 1. Contrarily, people with HAND did not differ from controls (*F*(3,50) = 1.271, *P* = 0.295). As for responses to Stimulation 2, people with probable Alzheimer’s disease exhibited a trending difference from controls (*F*(1,45) = 3.923, *P* = 0.054) and differed from people with HAND (*F*(3,44) = 3.426, *P* = 0.026), but again people with HAND did not differ from controls (*F*(3,50) = 0.330, *P* = 0.804). Finally, the stimulation main effect reflected stronger responses to Stimulation 1 relative to 2 across all groups (*F*(4,70) = 3.992, *P* = 0.050; Fig. 3).

To identify the impact of Alzheimer’s disease and HAND pathologies on somatosensory gating and be consistent with previous studies,^13,14,40^ we computed the gating ratio (i.e. response to Stimulation 2 divided by the response to Stimulation 1) and ran a 3 × 1 ANOVA. This indicated that there were no group differences in the gating ratio (*F*(4,70) = 0.222, *P* = 0.925).

### Increased spontaneous cortical activity in people with HAND

**Figure 4:**
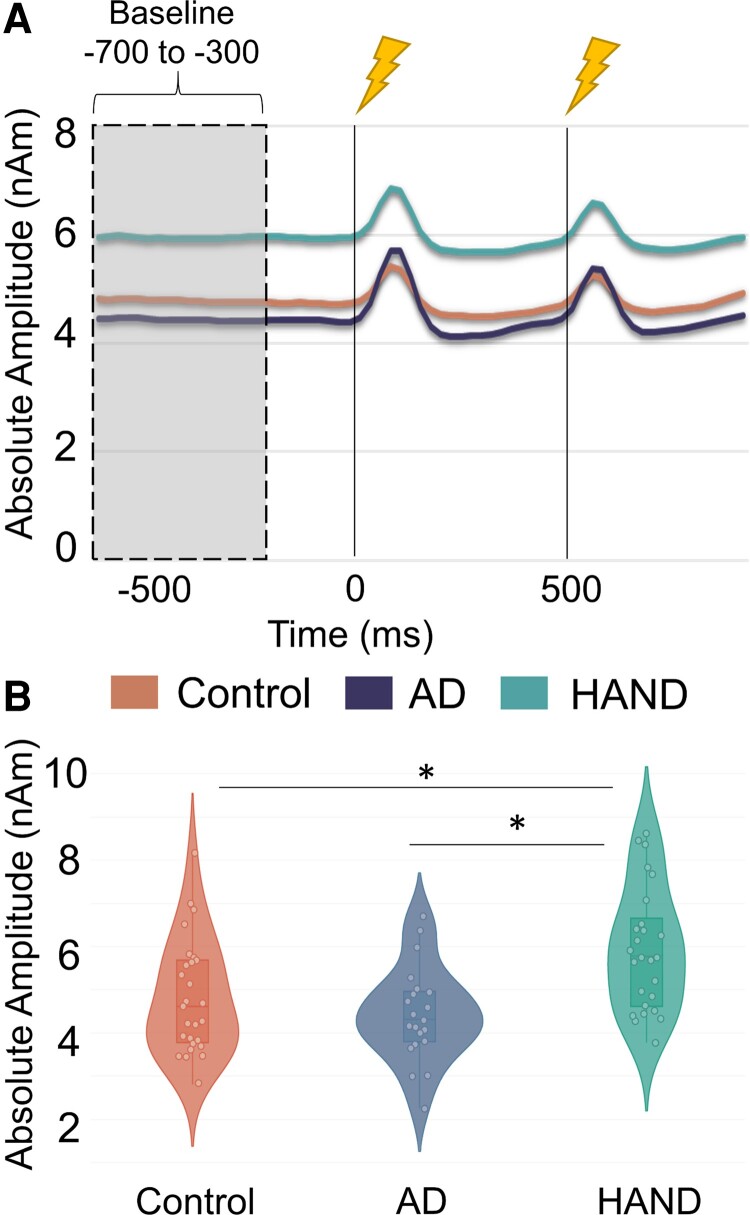
**Virtual sensor time series reveal group differences in spontaneous activity.** (**A**) Absolute amplitude time series showing the non-normalized amplitude as a function of time for the entire epoch in each group. The colour legend appears beneath the time series. As shown, persons with HAND had sharply elevated spontaneous gamma activity during the prestimulus baseline relative to controls and people with Alzheimer’s disease. The grey-shaded area reflects the baseline period (−700 to −300 ms) used to estimate the mean amplitude. The black lines at time = 0 ms and time = 500 ms indicate the onset of the paired-pulse electrical stimulation. (**B**) Violin plots showing the distribution of mean amplitude values during the prestimulus baseline period. All data points are shown along with the median and interquartile range. A 3 × 1 ANOVA (group × average spontaneous power during the baseline) suggested robust group differences. Follow-up *post hoc* analyses were conducted. *P < 0.05

To probe spontaneous activity, we computed the mean absolute amplitude during the prestimulus baseline period (i.e. −700 to −300 ms). A 3 × 1 ANOVA (group × average spontaneous power during the baseline) suggested robust group differences (*F*(4,70) = 5.060, *P* = 0.001). Follow-up *post hoc* analyses revealed this was driven by people with HAND, as they exhibited strongly elevated spontaneous activity relative to controls (*F*(3,50) = 4.465, *P* = 0.008) and people with Alzheimer’s disease (*F*(3,45) = 5.161, *P* = 0.004), while controls and people with Alzheimer’s disease did not differ from each other (*F*(1,47) = 1.003, *P* = 0.322; Fig. 4).

## Discussion

The goal of this study was to determine whether Alzheimer’s disease and HAND pathologies differentially affect cortical somatosensory processing and inhibitory function. Analysis of voxel time series data revealed robust somatosensory responses across a broad frequency range (20–75 Hz) following each stimulation of the right median nerve in all three groups, with the Alzheimer’s disease group exhibiting significantly stronger responses compared with both controls and those with HAND. Conversely, we observed significantly elevated prestimulus spontaneous cortical activity in those with HAND compared with both controls and the Alzheimer’s disease group. In other words, people with Alzheimer’s disease exhibited normal spontaneous neural activity prior to stimulus onset but elevated gamma oscillations in response to each stimulation, while people with HAND exhibited aberrantly elevated spontaneous power during the baseline period but normal gamma oscillations in response to each stimulation. Below, we discuss these novel findings and their implications for understanding the unique impact of Alzheimer’s disease and HAND pathologies on somatosensory function.

Previous studies have shown altered neural responses to visual^61–63^ and auditory stimulations^64,65^ in patients with Alzheimer’s disease. For example, in the visual cortex, multiple studies have shown decreased response amplitude in those with Alzheimer’s disease relative to controls,^66–68^ while similar observations have also emerged in the auditory cortex.^69,70^ Thus, the current findings extend observations of altered sensory responses in Alzheimer’s disease relative to controls to the somatosensory cortex, although unlike other sensory regions (i.e. auditory and visual) we found that patients with Alzheimer’s disease exhibited stronger responses in the somatosensory regions.^71^ As mentioned in the introduction, no studies to date have compared somatosensory processing in persons with HAND and Alzheimer’s disease, and the same is true for auditory, visual and other sensory cortices. Our finding that patients with Alzheimer’s disease exhibit stronger somatosensory responses than those with HAND is novel and supports the idea of distinct neuropathologies among the two diseases. Finally, our data suggested that patients with HAND exhibit similar responses to controls, which agrees with studies from our group and others,^13,15^ although studies have also shown reduced responses in persons with HIV compared with controls.^14,17^ Thus, future studies are warranted to decipher the clinical factors they may modulate this response in persons with HIV.

Another key finding from the current study was the elevated spontaneous amplitude observed during the prestimulus baseline period in persons with HAND. Such increased spontaneous activity has been previously reported in those with HAND relative to both controls and unimpaired PWH.^13–15,72,73^ While the aetiology of this increase in spontaneous activity has not been fully elucidated, it is speculated that this may reflect the accelerated aging exhibited by PWH,^39,74–78^ as older adults have also been shown to exhibit an increase in spontaneous neural activity in the somatosensory and motor cortices.^79–81^ Interestingly, people with Alzheimer’s disease did not have such elevations in spontaneous activity and, in fact, exhibited spontaneous activity levels in the left postcentral gyrus that were similar to controls. Thus, like the group differences in the relative neural responses to the somatosensory stimulations discussed above, our work suggests a fundamental difference between the impact of Alzheimer’s disease and HAND pathologies on somatosensory cortical function. Essentially, Alzheimer’s disease appears to primarily affect the gamma response to somatosensory stimulation, while HAND is associated with elevations in spontaneous cortical activity in the same brain tissue. Future studies should examine the cellular and molecular origins of these effects and whether they are specific to the somatosensory cortices. At least in the case of HAND, there is evidence that altered spontaneous activity extends to other brain regions.^15,72,73^

Finally, it should be noted that we did not find evidence supporting altered sensory gating in Alzheimer’s disease or HAND. In the case of Alzheimer’s disease, this is in disagreement with observations in the auditory cortex, which have shown that patients with Alzheimer’s disease exhibit impaired gating (i.e. reduced) relative to matched controls.^82,83^ Further, one recent study showed that such gating deficits can be obscured by differences in neurocognitive function.^40^ Interestingly, this latter study focused on somatosensory gating and involved patients across the Alzheimer’s disease spectrum (i.e. mild cognitive impairment (MCI) and mild Alzheimer’s disease). In the current study, our Alzheimer’s disease group scored comparably impaired on most domains of neurocognitive function and thus we did not attempt to covary these parameters out due to limited variance. Thus, future studies should consider enrolling patients with Alzheimer’s disease/MCI whose cognitive performance extends across a broader range and controlling for such differences when evaluating differences in sensory gating. Regarding HAND, our observation of normal gating in the somatosensory cortices also agrees with earlier studies.^13,14^

Before closing, it is important to acknowledge the limitations of the current study. First, our study was limited to the somatosensory cortices and future work should compare groups with Alzheimer’s disease and HAND on auditory and visual function. Second, our patients with HAND did not undergo amyloid-PET imaging to ascertain that they were in fact amyloid negative. However, numerous studies have now shown that patients with HAND are overwhelming amyloid negative,^84–88^ and thus not conducting PET imaging was a major cost savings and limited patient exposure to radiation. Third, the study was cross-sectional and future work would benefit from a longitudinal approach, as proteinopathies such as Alzheimer’s disease begin years prior to symptom onset and accumulate progressively throughout the disease course. Thus, neural parameters where our Alzheimer’s disease patients seemed to be normal (e.g. spontaneous gamma) may simply reflect their current disease stage. Fourth, our groups were not perfectly matched on age and education. Although these factors were used as covariates of no interest in all analyses, future studies should aim for more precise matching. Future studies should also consider exploring potential relationships between somatosensory processing metrics and neuropsychological data and/or demographic measures. Fifth, our sample size in the current study was only moderate and a reasonable future direction would be to replicate in a larger sample. Collecting measures of GABA concentration using magnetic resonance spectroscopy may also be fruitful in identifying mechanisms, and future studies should consider including such measures. Finally, due to the moderate sample size, we were unable to explore the impact of comorbid conditions known to be common in these populations or effects of medications. To this point, work has been conducted to suggest people with Alzheimer’s disease treated with cholinesterase inhibitors may have altered frequency-specific dynamics, particularly in the lower canonical frequency bands like delta and theta,^89,90^ which is distinct from the gamma responses we examined here, but nonetheless does suggest that more oscillatory-focused work should be done to understand the effects of medications in clinical populations to provide a fuller characterization of these findings.

## Conclusions

The current study examined the unique impact of Alzheimer’s disease and HAND neuropathologies on somatosensory function and inhibitory processing using a well-established paired-pulse somatosensory paradigm. Our data indicated that people with Alzheimer’s disease and HAND exhibit normal somatosensory gating but have aberrations in distinct elements of somatosensory cortical function. Essentially, those with Alzheimer’s disease exhibit accentuated responses to somatosensory stimulation along with normal spontaneous gamma preceding stimulus onset. Conversely, those with HAND exhibit normal responses to somatosensory stimulation along with sharply elevated spontaneous gamma activity prior to stimulus onset. Thus, these conditions are associated with unique aberrations in somatosensory cortical function, which could indicate the impact of different molecular mechanisms underlying the conditions. Further, given the differential pattern of deficits in somatosensory cortical function, these measures may function as unique markers of decline in each condition and be useful in identifying PWH who develop Alzheimer’s disease. Future studies should extend these findings to other sensory modalities and consider longitudinal approaches.

## Supplementary Material

fcac169_Supplementary_Data

## Abbreviations

cART =: combination antiretroviral therapy
HAD =: HIV-associated dementia
HAND =: HIV-associated neurocognitive disorder
MEG =: magnetoencephalography
PWH =: people with HIV or people with HAND
