# Supplementary material for: Signatures of somatosensory cortical dysfunction in Alzheimer’s disease and HIV-associated neurocognitive disorder
Source: Brain Commun. 2022 Jun 23;4(4):fcac169. doi: 10.1093/braincomms/fcac169 (PMC9260304; doi:10.1093/braincomms/fcac169)
Supplement: fcac169_Supplementary_Data [file fcac169_supplementary_data.docx]

Supplemental Materials

**Supplementary Table 1: MAMMOA Neuropsychological Test Z-Scores**

|  | **Controls (n = 14)** | **PWH (n = 26)** |
| --- | --- | --- |
| WRAT-4 | 0.39 [0.83] | -0.87 [0.95] |
| HVLT-R Total | 0.15 [0.64] | -1.7 [0.87] |
| HVLT-R Delayed Recall | 0.029 [0.85] | -1.7 [1.0] |
| HVLT-R Retention | -0.19 [0.77] | -0.92 [1.6] |
| HVLT-R Recognition  Discriminability Index | 0.36 [0.90] | -0.94 [1.2] |
| Trail Making Test- Part A | 0.61 [0.91] | -0.36 [0.93] |
| Trail Making Test- Part B | 0.54 [0.83] | -0.13 [0.67] |
| Grooved Pegboard - Dominant Hand | 0.14 [0.92] | -1.1 [0.94] |
| Grooved Pegboard -  Non-Dominant Hand | -0.0077 [0.68] | -1.3 [0.84] |
| Phonemic Verbal Fluency | 0.34 [0.92] | -0.57 [0.98] |
| Semantic Verbal Fluency | 0.26 [1.09] | -0.62 [1.09] |
| Digit Symbol | 1.5 [0.65] | -0.38 [0.62] |
| Digit Search | 1.4 [0.71] | -0.58 [0.82] |
| Stroop Color | 0.37 [0.88] | -0.97 [0.96] |
| Stroop Word | 0.19 [1.0] | -1.4 [1.0] |
| Stroop Interference | 0.27 [1.2] | -1.3 [1.6] |

Values are reported as mean [standard deviation]. Abbreviations: WRAT = Wide Range Achievement Test; HVLT = Hopkins Verbal Learning Test

**Supplementary Table 2: DMAP Neuropsychological Test Z-Scores**

|  | **Controls (n = 13)** | **PWH (n = 21)** |
| --- | --- | --- |
| WRAT-4 | 1.1 [0.70] | -0.17 [0.93] |
| MMSE7S | 29 [0.75] | 22 [3.9] |
| MMSE WORLD | 30 [0.66] | 22 [3.4] |
| WMS-IV Logical Memory I | 0.82 [0.82] | -2.2 [0.97] |
| WMS- IV Logical Memory II | 0.74 [1.1] | -2.6 [0.68] |
| WMS-IV Logical Memory Recognition | 0.57 [0.25] | -1.3 [0.89] |
| HVLT Total | 0.49 [0.97] | -2.7 [0.53] |
| HVLT Retention | -0.24 [0.63] | -3 [0.59] |
| HVLT Delayed Recall | 0.12 [0.83] | -3.0 [0.31] |
| HVLT-R Recognition  Discriminability Index | 0.046 [0.89] | -3.0 [0.61] |
| Trail Making Test- Part A | 0.52 [0.78] | -1.9 [1.5] |
| Trail Making Test- Part B | 1.1 [0.66] | -1.7 [1.2] |
| Boston Naming Test | 0.91 [1.2] | -0.64 [1.3] |
| FAS | -0.52 [0.83] | -1.3 [1.1] |
| Animals | -0.046 [1.0] | -2.6 [1.4] |
| MOCA | 27 [2.0] | 17 [4.1] |
| Digit Span Forward | 0.56 [0.96] | -0.57 [1.2] |
| Digit Span Backward | -0.14 [1.0] | -0.063 [0.91] |

Values are reported as mean [standard deviation]. Abbreviations: WRAT = Wide Range Achievement Test; MMSE = Mini Mental State Exam; WMS-IV = Weschler Memory Scale; HVLT = Hopkins Verbal Learning Test; MOCA = Montreal Cognitive Assessment.
